# Supplementary material for: Choice Architecture Cueing to Healthier Dietary Choices and Physical Activity at the Workplace: Implementation and Feasibility Evaluation
Source: Nutrients. 2021 Oct 14;13(10):3592. doi: 10.3390/nu13103592 (PMC8538928; doi:10.3390/nu13103592)
Supplement: Supplementary file 1 [file nutrients-13-03592-s001.zip › Supplementary material S1.pdf]

Supplementary material 1.

Toolkit for Creating Healthy Working Environments

This supplementary material portrays 1) the aims and evidence base and 2) the structure and content of the *StopDia Toolkit for Creating Healthy Working Environments*.

1. Aims and evidence base

The Toolkit comprises practical strategies for modifying physical and social working environments to facilitate healthier choices and the performance of small, healthy acts at the workplace within daily work tasks. The Toolkit applies dietary and physical activity recommendations, scientific literature, empirical knowledge from workplaces, and practical considerations relevant for the workplace setting.

The aim of the Toolkit is to support dietary and physical activity patterns recommended for promoting health and preventing non-communicable diseases and related risk factors [1–4] (**Table 1**).

Table 1. Aims of the Toolkit.

Higher level aim	Lower level aim
Healthy food choices ↑	Consumption of vegetables, fruit, berries, plain nuts, almonds, seeds ↑
	Drinking water ↑
	Drinking sugary beverages ↓
Regular meal pattern ↑	Having lunch or other main meal during a work shift ↑
	Having healthy snacks ↑
Physical activity ↑	Time spent sitting ↓
Sedentary behaviour ↓	Time spent standing ↑
	Amount of steps taken ↑
	Use of stairs ↑
	Short exercise bursts ↑
Recovery from work ↑	Taking breaks ↑

↑ = increases/strengthens, ↓ = decreases

The strategies of the Toolkit, their core components, and/or the mechanisms whereby they affect behaviour have proved effective in earlier scientific research. On a theoretical level, the Toolkit relies on the dual process theories of cognition, which assume that two types of cognitive processes, automatic and reflective, regulate decision-making and behaviour [5,6]. Striving to promote healthy behaviours, the Toolkit applies the nudge [7,8] and choice architecture [7,9] approaches. The idea of these approaches is to alter the choice architecture, i.e. the placement, presentation, arrangement, and properties of available choice options in the context or environment in which choices are made and the target behaviour takes place [7–9]. The core aim of nudge and choice architecture interventions is to facilitate choices and behaviours that serve the chooser's best interest, without limiting freedom of choice, significantly changing financial or other incentives, and relying on the provision of factual information or rational argumentation [7,8].

The Toolkit strategies employ numerous behaviour change mechanisms known to influence behaviour predominantly through automatic cognitive processes. The strategies were defined following three frameworks that support the application of these behavioural insights: TIPPME [10], MINDSPACE [11], and EAST [12]. The TIPPME Typology of Interventions in Proximal Physical Micro-Environments defines six intervention types and three spatial intervention foci; forming altogether 18 intervention categories [10]. The TIPPME interventions alter the placement (i.e. availability or position) or properties (i.e. functionality, presentation, size, or information) of objects and stimuli within small-scale micro-environments, targeting either products, product-related objects, or the wider environment [10]. The MINDSPACE [11] and EAST [12] frameworks serve as mnemonics and comprise nine and four behavioural approaches, respectively. The approaches of MINDSPACE are messenger, incentives, norms, defaults, salience, priming, affect, commitment, and ego. The approaches of EAST are easy, attractive, social, and timely.

Besides scientific literature, the strategies of the Toolkit consider interventions already executed at workplaces as well as the needs for and the challenges of workplace health promotion. These empirical data were collected during the development phase of the StopDia at Work intervention in stakeholder workshops (n = 4) and individual interviews (n = 23) involving representatives from 31 organisations. Finally, regarding

practical considerations, the strategies included in the Toolkit ought to be relevant for the workplace setting, applicable to various worksite environments, accessible to all employees of a workplace, and inexpensive and effortless as possible to implement and maintain.

2. Structure and content

Table 2 presents the practical strategies of the Toolkit, including suitable settings, ease of implementation, required purchases, applied behaviour change mechanisms, expected effects, and references to supporting evidence. The following paragraphs define the key concepts of the Toolkit.

2.1 Ease of implementation

Ease of implementation reflects the amount of knowledge and/or effort required to maintain a strategy after its launch on a tripartite scale: easy, moderate, and demanding. Easy-to-implement strategies require little specialised knowledge and besides occasional check-ups no maintenance after launch. Examples of such strategies are laying out posters and introducing new equipment or furniture. Moderate-to-implement strategies require some knowledge on correct implementation and light maintenance on a regular basis. Examples of such strategies are maintaining exercise equipment in pre-defined places, running a campaign that requires regular delivery of materials, and creating a social norm by reminding of a new, commonly agreed practice. Demanding-to-implement strategies require more specialised knowledge on correct implementation and daily maintenance. Examples of such strategies are the use of nutritional labels and placement of healthier foods in workplace cafeterias.

2.2 Required purchases

Required purchases suggestively indicate the extent to which implementation requires the procurement of new materials, goods, or services on a tripartite scale: none, minor, and substantial. None refers to strategies that require no procuring. Minor purchases refer to relatively inexpensive goods, such as gym sticks or water bottles, and substantial purchases to relatively expensive goods such as new furniture. Costs of purchases depend, however, on the price category of procured items and intervention dose delivered; for example, whether height-adjustable desks are provided for all employees or to common work environments only, or whether employees are provided fresh fruit every day or only once a week.

2.3 Behaviour change mechanisms

Behaviour change mechanisms portray how the Toolkit strategies can trigger changes in behaviour, and follow the above-described frameworks of TIPPME [10], MINDSPACE [11], and EAST [12].

2.4 Expected effects

Expected effects illustrate rough estimates of effect sizes on a tripartite scale: small, medium, and large. These estimates follow the findings and categorisation of a recent meta-analysis [13] that grouped nutrition-related nudge interventions into three categories: cognitively, affectively, and behaviourally oriented interventions. Cognitively oriented strategies influence primarily what people know, affectively oriented strategies how people feel, and behaviourally oriented strategies what people do. The meta-analysis found affectively oriented interventions more effective than cognitively oriented interventions, and behaviourally oriented interventions more effective than cognitively and affectively oriented interventions [13].

2.5 Healthy options

In this work, healthy options refer to food products, meals, and recipes that meet the nutritional criteria of the Heart Symbol*, the nutritional labelling system of the Finnish Heart Association and the Finnish Diabetes Association (https://www.sydanmerkki.fi/en/). Energy-free beverages, such as water, coffee, and tea count as healthy as well. The Heart Symbol is a nutritional claim according to EU regulation on nutrition and health claims made on foods (EC N° 1924/2006). A Heart Symbol-product represents nutritionally better a choice within its product category and meets category-specific criteria regarding fat (quantity and quality), salt, sugar, and fibre. These criteria build on the Finnish nutrition recommendations [2].

* The Heart Symbol. Image reproduced and used with the permission of The Finnish Heart Association.

Table 2. The StopDia Toolkit for Creating Healthy Working Environments.

Target		Practical strategy	Workplace setting	Ease of implementation	Required purchases	Behaviour change mechanism [1]	Expected effect	Reference
Nutrition		***Food provision***						
	1.	Enable healthy food and beverage choices by making them available at the workplace.	Cafeteria, vending machines, coffee rooms, events	Moderate	Minor	Product availability ↑ [T]	Small	[14]
	2.	Widen the selection and proportion of healthy food and beverage options available at the workplace.	Cafeteria, vending machines, events	Moderate	Minor	Product availability ↑ [T]<br>Attractive (salience ↑) [M,E]	Small	[14–19]
	3.	Replace energy dense and nutritionally poor food and beverage options with similar but nutritionally better alternatives.	Cafeteria, vending machines, coffee rooms, events	Moderate	Minor	Product availability ↑[T]<br>Easy (substitution, default) [E]	Large	[14]
	4.	Introduce snack spots in which healthy snacks and beverages are available for employees.	Common work environments	Moderate	Minor	Product availability ↑ [T]	Small	[14,15,20,21]
	5.	Place healthy options according to the following principles:<br>a.) on visible, easily noticeable spots<br>b.) at the beginning of the buffet<br>c.) closer to the chooser (e.g., in front row)<br>d.) in the middle of the tray, shelf, or showcase	Cafeteria, vending machines, events	Demanding	None	Product position [T]<br>Easy (friction costs ↓) [E]<br>Attractive (salience ↑) [M, E]	Small	[14,17,21–27]
	6.	Place less healthy options according to the following principles:<br>a.) on less visible and easily noticeable spots<br>b.) at the end of the buffet<br>c.) further away from the chooser (e.g., in back row)<br>d.) on the edge of the tray, shelf, or showcase	Cafeteria, vending machines, events	Demanding	None	Product position [T]<br>Less easy (friction costs ↑) [E]<br>Less attractive (salience ↓) [M,E]	Small	[14,17,21–27]
	7.	Place healthy options first on menus.	Cafeteria	Moderate	None	Related object position [T]<br>Easy (ordering effect) [E]<br>Attractive (salience ↑) [M,E]	Small	[14,28,29]
	8.	To avoid constant "grazing" at workplace events such as meetings, place provided foods and beverages so that accessing them requires standing up and taking a couple of steps.	Events	Moderate	None	Product position [T]<br>Less easy (friction costs ↑, default) [M,E]	Large	[14,21]
	9.	To increase convenience, serve fruit and vegetable ready to eat, i.e., washed, peeled if needed, and cut into pieces.	Cafeteria, coffee rooms, events	Demanding	None	Product functionality [T]<br>Easy (friction costs ↓) [E]	Large	[13,30]
	10.	To increase perceived variety and encourage greater consumption, serve various fruit and vegetable varieties from separate serving dishes instead of mixing them together.	Cafeteria, coffee rooms, events	Moderate	None	Product presentation [T]<br>Attractive (salience ↑) [M,E]	Small	[22]
	11.	Serve healthy options, such as fruit and vegetable temptingly using attractive displays and serving dishes.	Cafeteria, coffee rooms, events	Moderate	None	Product and related object presentation [T]<br>Attractive (salience ↑) [M,E]	Medium	[13,31]
	12.	Guide employees to healthy options with bright-coloured directional signs, such as footprints, tapes, or arrows on the floor or walls.	Cafeteria	Easy	Minor	Atmospheric properties of the wider environment [T]<br>Attractive (salience ↑) [M,E]	Small	[27]
	13.	Light healthy options brightly.	Cafeteria	Easy	Substantial	Atmospheric properties of the wider environment [T]<br>Attractive (salience ↑) [M,E]	Small	[32,33]

Target	Practical strategy	Workplace setting	Ease of implementation	Required purchases	Behaviour change mechanism [1]	Expected effect	Reference
	14. Where unhealthy foods and beverages are served, use reflecting surfaces such as mirrors to strengthen employee self-awareness.	Cafeteria, vending machines	Easy	Substantial	Atmospheric properties of the wider environment [T]<br>Ego (maintain positive self-image) [M]	Small	[34,35]
	15. Use larger serving dishes for fruit and vegetable.	Cafeteria, events	Moderate	None	Product size [T]<br>Easy (default) [M,E]	Large	[13,36]
	16. Use smaller serving dishes for less healthy options.	Cafeteria, events	Moderate	None	Product size [T]<br>Easy (default) [M,E]	Large	[13,36]
	17. Use larger plates for salad.	Cafeteria, events	Moderate	None	Product size [T]<br>Easy (default) [M,E]	Large	[13,36]
	18. Use smaller plates and bowls for main courses and desserts	Cafeteria, events	Moderate	None	Product size [T]<br>Easy (default) [M,E]	Large	[13,36]
	19. User larger tongs and spoons for fruit and vegetable	Cafeteria, events	Moderate	None	Product size [T]<br>Easy (default) [M,E]	Large	[13,26,36]
	20. Use smaller tongs and spoons for less healthy options	Cafeteria, events	Moderate	None	Product size [T]<br>Less easy (default) [M,E]	Large	[13,26,36]
	21. Use larger serving sizes for fruit and vegetables	Cafeteria, events	Moderate	None	Product size [T]<br>Easy (default) [M,E]	Large	[13,36]
	22. Use smaller serving sizes for less healthy options	Cafeteria, events	Moderate	None	Product size [T]<br>Easy (default) [M,E]	Large	[13,30,36]
	23. Adopt a "one plate-policy" at workplace cafeteria; move separate bread and salad plates out of sight to guide employees choose one large plate; thus facilitating the composition of the meal according to the plate model (i.e., 1/2 vegetable, 1/4 protein, and 1/4 carbohydrates). For the strategy to be effective, salads should be placed first in the buffet line.	Cafeteria	Easy	None	Product size [T]<br>Easy (default) [M,E]	Large	[13,36]
	24. Indicate healthy options with the Heart Symbol on menus and at the point-of-choice.	Cafeteria, vending machines	Demanding	Minor	Information on related objects [T]<br>Attractive (salience ↑) [M,E]<br>Timely (prompting) [E]<br>Easy (simplification) [E]	Small	[13,23–25,27,37–39]
	25. Use *Follow the heart*-posters [2] at restaurant entrance and/or at the beginning of the buffet to guide customers notice and choose options labelled with the Heart Symbol.	Cafeteria	Easy	None	Information within the wider environment [T]<br>Attractive (salience ↑) [M,E]<br>Timely (priming) [M,E]	Small	[27,39]
	26. Name healthy options temptingly, for example, describing their taste, texture, or look.	Cafeteria	Moderate	None	Information on related objects [T]<br>Attractive (salience ↑) [M,E]<br>Affect [M]<br>Timely (priming) [M,E]	Medium	[13,40,41]
	Drinking water						
	27. Use larger glasses for water.	Cafeteria, events	Moderate	None	Product size [T]<br>Easy (default) [M,E]	Large	[13,36]
	28. Provide personal, reusable water bottles or mugs for all employees to facilitate and remind of drinking water.	Personal workstation	Easy	Minor	Related object availability ↑ [T]<br>Easy (friction costs ↓) [E]	Small	[21,42]

Target		Practical strategy	Workplace setting	Ease of implementation	Required purchases	Behaviour change mechanism [1]	Expected effect	Reference
	29.	Place water coolers or pitchers and water glasses on easily noticeable spots at the workplace, for example, along common passing routes or where employees typically pause for a moment.	Common work environments	Demanding	Minor	Product and related object availability ↑ [T]<br>Attractive (salience ↑) [M,E]	Small	[14,23,24,42]
		Packed lunches and snacks						
	30.	Promote and share temptingly named, visually attractive, and seasonal *StopDia Packed Lunch of the Week*-recipes [3] at workplace coffee rooms and/or via electronic channels, such as info-screens, company intranet, and newsletters. The campaign comprises one recipe for each week of the year, and all recipes meet the nutritional criteria of the Heart Symbol.	Coffee rooms	Moderate	None	Easy (friction costs ↓, chunking) [E]<br>Attractive (salience ↑) [M,E]<br>Social (descriptive norm) [M,E]<br>Timely (priming) [M,E]<br>Affect [M]	Small	[13,41,43]
	31.	Promote and provide employees the *Fruit Crew*-starting kit [4] that facilitates colleagues to found a fruit circle and consequently have fresh fruit available at the workplace.	Coffee rooms	Easy	None	Social (network nudge, commitment contracts, descriptive norm, reciprocity) [M,E]<br>Attractive (gamification, salience ↑) [M,E]<br>Timely (implementation intentions) [E]	Small	[13,43–45]
	32.	Make cold storage of packed lunches possible for travelling employees, for example, by providing a cool bag, thus facilitating healthy meals, maintenance of a regular meal pattern, and enjoying packed lunches fresh while travelling.	Personal workstation	Easy	Minor	Related object availability ↑ [T]<br>Easy (friction costs ↓) [E]	Small	[46,47]
Physical activity		***Time spent sitting***						
	33.	Introduce height-adjustable desks to enable working by standing.	Personal and common work environments	Easy	Substantial	Product availability [T]<br>Easy (friction costs ↓) [E]	Small	[48,49]
	34.	Make working by standing the default option, for example, by commonly agreeing on a practice of leaving height-adjustable desks in the upper position at the end of the workday.	Common work environments	Moderate	None	Easy (default) [M,E]<br>Social norm [M,E]	Large	[13,50,51]
	35.	Introduce alternative seats, such as therapy balls, saddle or wobble chairs, or balance cushions to enable active sitting.	Personal and common work environments	Easy	Substantial	Product availability [T]<br>Easy (friction costs ↓) [E]	Small	[48,49,52]
	36.	Make active sitting the default option by placing available alternative seats, such as therapy balls, saddle or wobble chairs, or balance cushions in front of workstations.	Common work environments	Moderate	None	Easy (default) [M,E]	Large	[13,50]
	37.	Enable physical activity during meetings by 1) arranging enough room for standing up, moving around, and stretching; and 2) introducing height-adjustable desks, alternative seats (e.g., therapy balls, saddle or wobble chairs, exercise bikes, or balance cushions), and pads or wheels under chair feet so that moving chairs and standing up can be done silently without disturbance.	Meeting rooms	Easy	1) None<br>2) Substantial	Product and wider environment availability [T]<br>Easy (friction costs ↓) [E]	Small	[48,49,52]
	38.	Create "walking meeting" as a meeting option in online calendars. By booking, the system could suggest an appropriate-length walking route near the workplace.	Personal workstation	Easy	Substantial	Product availability [T]<br>Easy (friction costs ↓) [E]<br>Timely (prompting) [E]<br>Ego (maintain positive self-image) [M]	Small	[48,49]

Target	Practical strategy	Workplace setting	Ease of implementation	Required purchases	Behaviour change mechanism [1]	Expected effect	Reference
	39. Replace personal printers with shared copy machines, or move personal printers from within reach to a distance that requires standing up and taking a few steps.	Common work environments	Easy	Substantial (or none)	Product position [T]<br>Easy (default) [M,E]	Large	[13,48,49,53]
	40. Make physical activity a social norm. While opening the meeting, the chairperson encourages everyone to stand up, walk, and take break exercise whenever they feel like it, and follows the given recommendations him-/herself.	Meetings	Moderate	None	Social norm [M,E]<br>Timely (prompting) [E]	Medium	[43,53,54]
	41. Commonly agree on an organisational practice of walking to talk to colleagues instead of calling or sending e-mail.	All work environments	Moderate	None	Social norm [M,E]	Medium	[43,48,53]
	Stair use						
	42. Keep stairwell doors open for easy access.	Stairs	Easy	None	Product functionality [T]<br>Easy (friction costs ↓) [E]<br>Attractive (salience ↑) [M,E]	Small	[55]
	43. Slow down elevator doors or the elevator itself to encourage choosing the stairs.	Elevator	Easy	Minor	Product functionality [T]<br>Easy (friction costs ↓) [E]	Large	[56]
	44. Enhance stairwell visibility with motivational and/or directional signs, for example, footprints on the floor leading to stairs from the point-of-choice between the stairs and the elevator or escalator.	Elevator, escalator, stairs	Easy	Minor	Atmospheric properties of the wider environment [T]<br>Attractive (salience ↑) [M,E]<br>Timely (prompting) [E]	Small	[52,55,57–60]
	45. Enhance stairwell attractiveness with, for example, decoration, artwork, plants, or lighting.	Stairs	Easy	Minor	Atmospheric properties of the wider environment [T]<br>Attractive (salience ↑) [M,E]<br>Affect [M]	Medium	[55,57,59–61]
	46. Create an attractive sound scape in the stairwell, for example, with calming nature sounds (e.g., ocean waves or rain) or pleasant music.	Stairs	Moderate	Substantial	Atmospheric properties of the wider environment [T]<br>Attractive (salience ↑) [M,E]<br>Affect[2]	Medium	[57,62]
	47. Introduce a captivating message, such as a story, a riddle, or a poem that leads to the stairs from the point-of-choice between the stairs and the elevator or escalator.	Elevator, escalator, stairs	Easy	Minor	Timely (prompting) [E]<br>Attractive (salience ↑) [M,E]<br>Affect[2]	Small	[57,61]
	48. Place stickers with the StopDia project logo [5] on elevator doors, next to the call buttons, or in their immediacy.	Elevator	Easy	Minor	Timely (prompting) [E]	Small	[57,60]
	Movement breaks						
	49. Place StopDia *Flex!*-movement posters [6] on salient spots where employees typically pause for a moment and have the opportunity to perform a movement or two. Such spots can be, for example, by copy machines, micros, or coffee makers.	Common work environments	Easy	None	Timely (prompting) [E]<br>Attractive (salience ↑) [M,E]<br>Easy (chunking) [E]	Small	[43,48,63–69]
	50. Provide light exercise equipment, such as gym sticks, balance boards, or hanging bars for employees to use.	Common work environments	Easy	Minor	Product availability [T]<br>Easy (friction costs ↓) [E]	Small	[35,49]
	51. Place available exercise equipment on salient spots where employees typically pause for a moment, and an opportunity for a short exercise break occurs. Such spots can be, for example, by copy machines, micros, kettles, or coffee makers.	Common work environments	Moderate	None	Timely (prompting) [E]<br>Attractive (salience ↑) [M,E]	Small	[35]

Target		Practical strategy	Workplace setting	Ease of implementation	Required purchases	Behaviour change mechanism [1]	Expected effect	Reference
	52.	Introduce an application that prompts employees to stand up, walk, or take short exercise breaks at pre-set intervals, for example, once every 1–2 hours.	Personal workstation	Easy	Minor	Timely (prompting) [E]	Small	[48,70–74]
Recovery from work		***Recovery***						
	53.	Introduce a silent space dedicated for relaxation and recovery from work.	Common work environments	Easy	None	Wider environment availability [T]	Small	[75]

[1] Behaviour change mechanisms: T = TIPPME [10], M = MINDSPACE [11], E = EAST [12]; ↑ = increase, ↓ = decrease

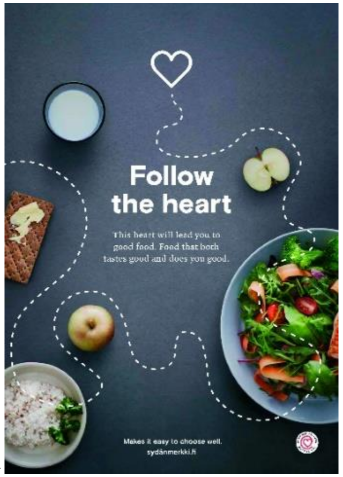

2

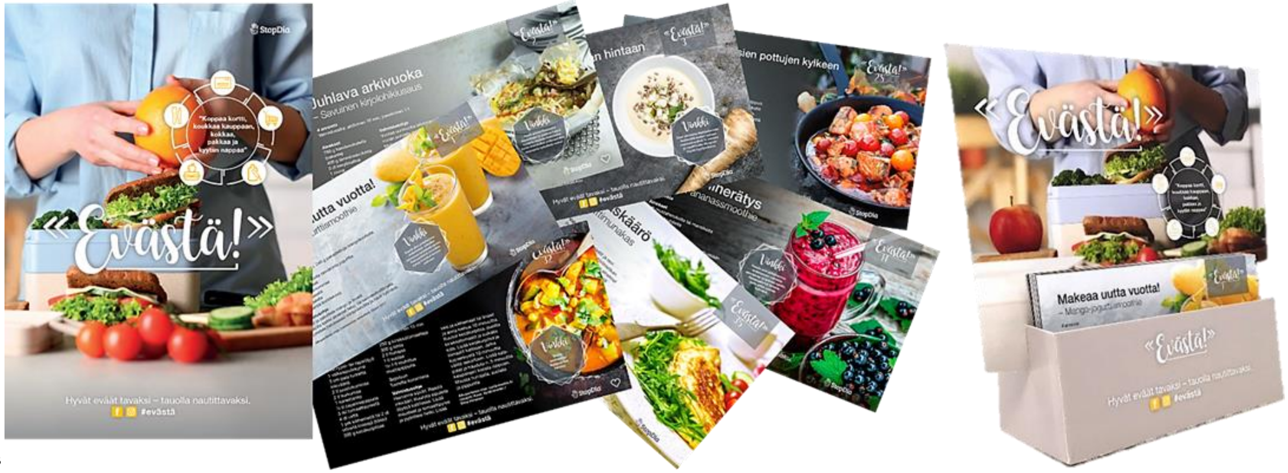

3

[2] *Follow the heart*-poster saying: "This heart will lead you to good food. Food that both tastes good and does you good." (sizes A4 and A3). Image reproduced and used with the permission of The Finnish Heart Association.

[3] StopDia Packed Lunch of the Week-materials: campaign poster (size A2), 52 various recipes (printed recipe card size 210 x 120 mm), and a cardboard stand for the recipe cards (width 285 mm, height 280 mm). The poster and the recipes were available in printed and/or electronic format. The poster and the stand featured a slogan that encouraged making a habit of enjoying a good packed lunch on a break, and a rhyme that prompted to pick up a recipe card, stop by the store, and prepare, pack, and grab the packed lunch.

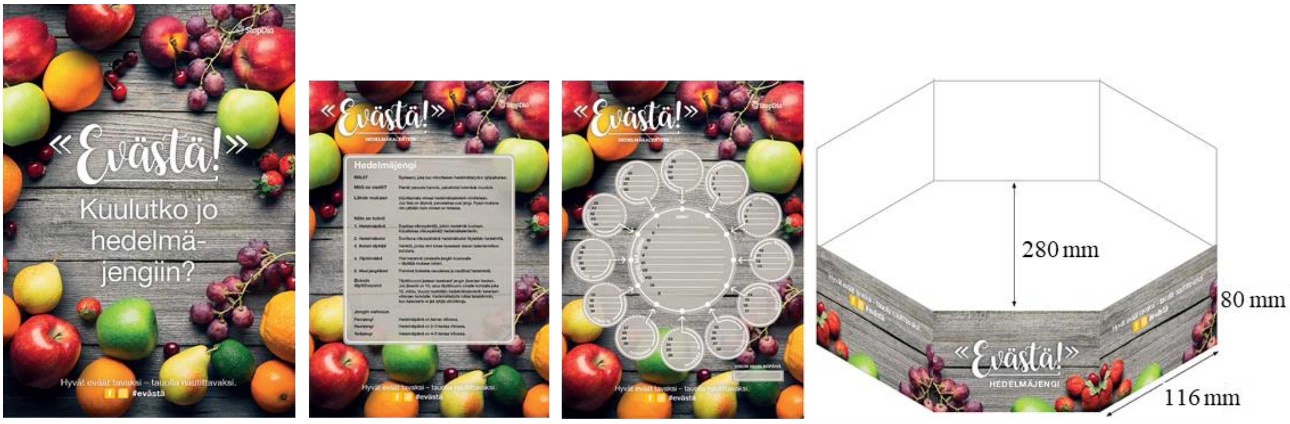

[4] StopDia Fruit Crew-materials: a poster asking "Have you joined a fruit crew already?" (size A2), instructions and enrolment form (size A4, two-sided), and a recyclable cardboard box for fruit. The poster was available in printed and/or electronic format.

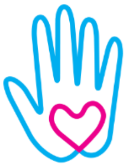

[5] The StopDia-logo (sticker size 105 x 150 mm)

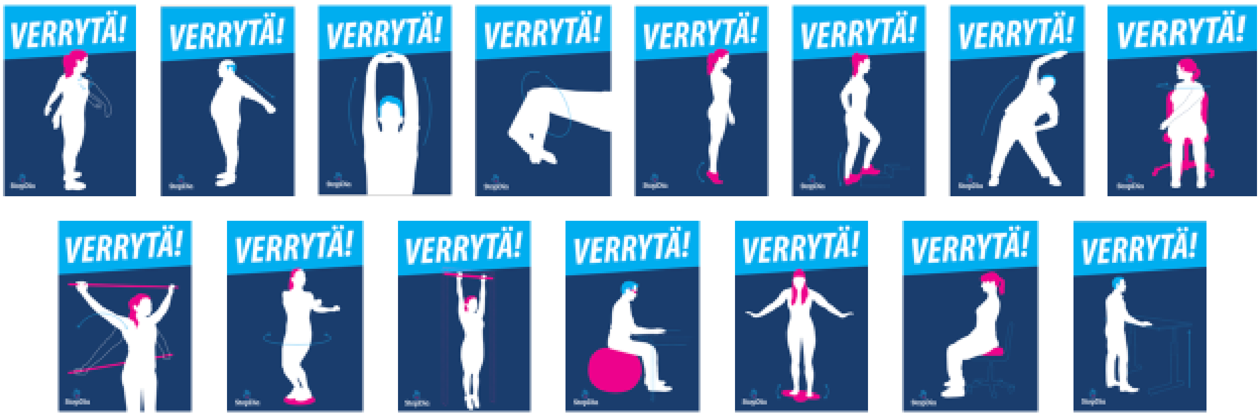

[6] StopDia *Flex!*-movement pictures available in printed (sizes A6, A5, and A4) and/or electronic format.

References

1. Nordic Council of Ministers *Nordic Nutrition Recommendations 2012: Integrating Nutrition and Physical Activity*; 5th ed.; Nordic Council of Ministers: Copenhagen, 2014; ISBN 9789289326704.
2. The National Nutrition Council of Finland *The Finnish Nutrition Recommendations 2014*; 4th ed.; The National Nutrition Council of Finland: Helsinki, 2014; ISBN ISBN 978-952-453-801-5.
3. U.S. Department of Health and Human Services *Physical Activity Guidelines for Americans*; 2nd ed.; U.S. Department of Health and Human Services: Washington DC, 2018.
4. UKK-instituutti. *Liikkumalla Terveyttä - Askel Kerrallaan. Viikoittainen Liikkumisen Suositus 18-64-Vuotiaille.* UKK-instituutti: Tampere, 2019. Available online: https://ukkinstituutti.fi/liikkuminen/liikkumisen-suositukset/aikuisten-liikkumisen-suositus/ (accessed on 27 April 2021).
5. Strack, F.; Deutsch, R. Reflective and Impulsive Determinants of Social Behavior. *Personality and Social Psychology Review* **2004**, *8*, 220–247.
6. Hofmann, W.; Friese, M.; Wiers, R.W. Impulsive versus Reflective Influences on Health Behavior: A Theoretical Framework and Empirical Review. *Health Psychology Review* **2008**, *2*, 111–137, doi:10.1080/17437190802617668.

7. Thaler, R.H.; Sunstein, C.R. *Nudge: Improving Decisions about Health, Wealth, and Happiness*; Updated ed.; Penguin Books: London, 2009.
8. Hansen, P.G. The Definition of Nudge and Libertarian Paternalism: Does the Hand Fit the Glove? *European Journal of Risk Regulation* **2016**, *7*, 155–174, doi:10.1017/S1867299X00005468.
9. Hollands, G.J.; Shemilt, I.; Marteau, T.M.; Jebb, S.A.; Kelly, M.P.; Nakamura, R.; Suhrcke, M.; Ogilvie, D. Altering Micro-Environments to Change Population Health Behaviour: Towards an Evidence Base for Choice Architecture Interventions. *BMC Public Health* **2013**, *13*, 1218–1223, doi:10.1186/1471-2458-13-1218.
10. Hollands, G.J.; Bignardi, G.; Johnston, M.; Kelly, M.P.; Ogilvie, D.; Petticrew, M.; Prestwich, A.; Shemilt, I.; Sutton, S.; Marteau, T.M. The TIPPME Intervention Typology for Changing Environments to Change Behaviour. *Nature Human Behaviour* **2017**, *1*, 1–9, doi:10.1038/s41562-017-0140.
11. Dolan, P.; Hallsworth, M.; Halpern, D.; King, D.; Metcalfe, R.; Vlaev, I. Influencing Behaviour: The Mindspace Way. *Journal of Economic Psychology* **2012**, *33*, 264–277, doi:10.1016/j.joep.2011.10.009.
12. Service, O.; Hallsworth, M.; Halpern, D.; Algate, F.; Gallagher, R.; Nguyen, S.; Ruda, S.; Sanders, M.; Pelenur, M.; Gyani, A.; et al. *EAST - Four Simple Ways to Apply Behavioural Insights*; The Behavioural Insights Team, Cabinet Office: London, 2016.
13. Cadario, R.; Chandon, P. Which Healthy Eating Nudges Work Best? A Meta-Analysis of Field Experiments. *Marketing Science* **2019**, doi:10.1287/mksc.2018.1128.
14. Hollands, G.J.; Carter, P.; Shemilt, I.; Marteau, T.M.; Jebb, S.A.; Higgins, J.; Ogilvie, D. Altering the Availability or Proximity of Food, Alcohol and Tobacco Products to Change Their Selection and Consumption. *Cochrane Database of Systematic Reviews* **2019**, CD012573, doi:10.1002/14651858.CD012573.
15. Rosi, A.; Zerbini, C.; Pellegrini, N.; Scazzina, F.; Brighenti, F.; Lugli, G. How to Improve Food Choices through Vending Machines: The Importance of Healthy Food Availability and Consumers' Awareness. *Food Quality and Preference* **2017**, *62*, 262–269, doi:10.1016/j.foodqual.2017.05.008.
16. Van Kleef, E.; Otten, K.; Van Trijp, H.C.M. Healthy Snacks at the Checkout Counter: A Lab and Field Study on the Impact of Shelf Arrangement and Assortment Structure on Consumer Choices. *BMC Public Health* **2012**, *12*, 1072–1082, doi:10.1186/1471-2458-12-1072.
17. Velema, E.; Vyth, E.L.; Hoekstra, T.; Steenhuis, I.H. Nudging and Social Marketing Techniques Encourage Employees to Make Healthier Food Choices: A Randomized Controlled Trial in 30 Worksite Cafeterias in The Netherlands. *The American Journal of Clinical Nutrition* **2018**, *107*, 236–246, doi:10.1093/ajcn/nqx045.
18. Hanks, A.S.; Just, D.R.; Smith, L.E.; Wansink, B. Healthy Convenience: Nudging Students toward Healthier Choices in the Lunchroom. *Journal of Public Health* **2012**, *34*, 370–376, doi:10.1093/pubmed/fds003.
19. Cullen, K.W.; Hartstein, J.; Reynolds, K.D.; Vu, M.; Resnicow, K.; Greene, N.; White, M.A. Improving the School Food Environment: Results from a Pilot Study in Middle Schools. *Journal of American Dietetic Association* **2007**, *107*, 484–489, doi:10.1016/j.jada.2006.12.004.
20. Baskin, E.; Gorlin, M.; Chance, Z.; Novemsky, N.; Dhar, R.; Huskey, K.; Hatzis, M. Proximity of Snacks to Beverages Increases Food Consumption in the Workplace: A Field Study. *Appetite* **2016**, *103*, 244–248, doi:10.1016/j.appet.2016.04.025.
21. Bucher, T.; Collins, C.; Rollo, M.E.; McCaffrey, T.A.; de Vlieger, N.; van der Bend, D.; Truby, H.; Perez-Cueto, F.J.A. Nudging Consumers towards Healthier Choices: A Systematic Review of Positional Influences on Food Choice. *British Journal of Nutrition* **2016**, *115*, 2252–2263, doi:10.1017/S0007114516001653.
22. Kongsbak, I.; Skov, L.R.; Nielsen, B.K.; Ahlmann, F.K.; Schaldemose, H.; Atkinson, L.; Wichmann, M.; Pérez-Cueto, F.J.A. Increasing Fruit and Vegetable Intake among Male University Students in an Ad Libitum Buffet Setting: A Choice Architectural Nudge Intervention. *Food Quality and Preference* **2016**, *49*, 183–188, doi:10.1016/j.foodqual.2015.12.006.
23. Thorndike, A.N.; Riis, J.; Sonnenberg, L.M.; Levy, D.E. Traffic-Light Labels and Choice Architecture: Promoting Healthy Food Choices. *American Journal of Preventive Medicine* **2014**, *46*, 143–149, doi:10.1016/j.amepre.2013.10.002.
24. Thorndike, A.N.; Sonnenberg, L.; Riis, J.; Barraclough, S.; Levy, D.E. A 2-Phase Labeling and Choice Architecture Intervention to Improve Healthy Food and Beverage Choices. *American Journal of Public Health* **2012**, *102*, 527–533, doi:10.2105/AJPH.2011.300391.
25. Levy, D.E.; Riis, J.; Sonnenberg, L.M.; Barraclough, S.J.; Thorndike, A.N. Food Choices of Minority and Low-Income Employees: A Cafeteria Intervention. *American Journal of Preventive Medicine* **2012**, *43*, 240–248, doi:10.1016/j.amepre.2012.05.004.
26. Rozin, P.; Scott, S.; Dingley, M.; Urbanek, J.K.; Jiang, H.; Kaltenbach, M. Nudge to Nobesity I: Minor Changes in Accessibility Decrease Food Intake. *Judgment and Decision Making* **2011**, *6*, 323–332, doi:10.1111/j.1753-4887.2009.00206.x.
27. Carins, J.E.; Rundle-Thiele, S.R.; Parkinson, J.E. Delivering Healthy Food Choice: A Dual-Process Model Enquiry. *Social Marketing Quarterly* **2017**, *23*, 266–283, doi:10.1177/1524500417709767.

28. Dayan, E.; Bar-Hillel, M. Nudge to Nobesity II: Menu Positions Influence Food Orders. *Judgment and Decision Making* **2011**, *6*, 333–342.
29. Wisdom, J.; Downs, J.S.; Loewenstein, G.; Burakowski, L.; Chaoka, A.; Fitzgerald, C.; Gales, N.; Haldane, L.; Park, M.Y.; Tang, E.; et al. Promoting Healthy Choices: Information versus Convenience. *American Economic Journal: Applied Economics* **2010**, *2*, 164–178, doi:10.1257/app.
30. Hansen, P.G.; Skov, L.R.; Jespersen, A.M.; Skov, K.L.; Schmidt, K. Apples versus Brownies: A Field Experiment in Rearranging Conference Snacking Buffets to Reduce Short-Term Energy Intake. *Journal of Foodservice Business Research* **2016**, *19*, 122–130, doi:10.1080/15378020.2016.1129227.
31. Ensaff, H.; Homer, M.; Sahota, P.; Braybrook, D.; Coan, S.; McLeod, H. Food Choice Architecture: An Intervention in a Secondary School and Its Impact on Students' Plant-Based Food Choices. *Nutrients* **2015**, *7*, 4426–4437, doi:10.3390/nu7064426.
32. Biswas, D.; Szocs, C.; Chacko, R.; Wansink, B. Shining Light on Atmospherics: How Ambient Light Influences Food Choices. *Journal of Marketing Research* **2017**, *54*, 111–123, doi:10.1509/jmr.14.0115.
33. Stroebele, N.; De Castro, J.M. Effect of Ambience on Food Intake and Food Choice. *Nutrition* **2004**, *20*, 821–838, doi:10.1016/j.nut.2004.05.012.
34. Beaman, A.L.; Klentz, B.; Diener, E.; Svanum, S. Self-Awareness and Transgression in Children: Two Field Studies. *Journal of Personality and Social Psychology* **1979**, *37*, 1835–1846, doi:10.1037/0022-3514.37.10.1835.
35. Papies, E.K. Health Goal Priming as a Situated Intervention Tool: How to Benefit from Nonconscious Motivational Routes to Health Behaviour. *Health Psychology Review* **2016**, *10*, 408–424, doi:10.1080/17437199.2016.1183506.
36. Hollands, G.J.; Shemilt, I.; Marteau, T.M.; Jebb, S.A.; Lewis, H.B.; Wei, Y.; Higgins, J.P.T.; Ogilvie, D. Portion, Package or Tableware Size for Changing Selection and Consumption of Food, Alcohol and Tobacco. *The Cochrane database of systematic reviews* **2015**, CD011045, doi:10.1002/14651858.CD011045.pub2.
37. Filimonau, V.; Lemmer, C.; Marshall, D.; Bejjani, G. "Nudging" as an Architect of More Responsible Consumer Choice in Food Service Provision: The Role of Restaurant Menu Design. *Journal of Cleaner Production* **2017**, *144*, 161–170, doi:10.1016/j.jclepro.2017.01.010.
38. Wilson, A.L.; Buckley, E.; Buckley, J.D.; Bogomolova, S. Nudging Healthier Food and Beverage Choices through Salience and Priming. Evidence from a Systematic Review. *Food Quality and Preference* **2016**, *51*, 47–64, doi:10.1016/j.foodqual.2016.02.009.
39. Levin, S. Pilot Study of a Cafeteria Program Relying Primarily on Symbols to Promote Healthy Choices. *Journal of Nutrition Education* **1996**, *28*, 282–285, doi:10.1016/S0022-3182(96)70102-4.
40. Grabenhorst, F.; Schulte, F.P.; Maderwald, S.; Brand, M. Food Labels Promote Healthy Choices by a Decision Bias in the Amygdala. *NeuroImage* **2013**, *74*, 152–163, doi:10.1016/j.neuroimage.2013.02.012.
41. Turnwald, B.P.; Boles, D.Z.; Crum, A.J. Association between Indulgent Descriptions and Vegetable Consumption: Twisted Carrots and Dynamite Beets. *JAMA Internal Medicine* **2017**, *177*, 1216–1218, doi:10.1001/jamainternmed.2017.1630.
42. Engell, D.; Kramer, M.; Malafi, T.; Salomon, M.; Lesher, L. Effects of Effort and Social Modeling on Drinking in Humans. *Appetite* **1996**, *26*, 129–138, doi:10.1006/appe.1996.0011.
43. Burke, M.A.; Peyton-Young, H. Social Norms. In *The Handbook of Social Economics*; Bisin, A., Benhabib, J., Jackson, M., Eds.; North Holland: Amsterdam, 2011; pp. 311–338.
44. Cialdini, R.B. *Influence: The Psychology of Persuasion*; Revised ed.; HarperBusiness: New York, 2007;
45. Hummel, D.; Maedche, A. How Effective Is Nudging? A Quantitative Review on the Effect Sizes and Limits of Empirical Nudging Studies. *Journal of Behavioral and Experimental Economics* **2019**, *80*, 47–58, doi:10.1016/j.socec.2019.03.005.
46. Hollands, G.J.; Shemilt, I.; Marteau, T.M.; Jebb, S.A.; Kelly, M.P.; Nakamura, R.; Suhrcke, M.; Ogilvie, D. *Altering Choice Architecture to Change Population Health Behaviour: A Large-Scale Conceptual and Empirical Scoping Review of Interventions within Micro-Environments*; University of Cambridge: Cambridge, 2013.
47. Hollands, G.J.; Bignardi, G.; Johnston, M.; Kelly, M.P.; Ogilvie, D.; Petticrew, M.; Prestwich, A.; Shemilt, I.; Sutton, S.; Marteau, T.M. The TIPPME Intervention Typology for Changing Environments to Change Behaviour. Supplementary Information. *Nature Human Behaviour* **2017**, *1*, 1-9. Suppl 1:1–5, doi:10.1038/s41562-017-0140.
48. Shrestha, N.; Kukkonen-Harjula, K.; Verbeek, J.; Ijaz, S.; Hermans, V.; Pedisic, Z. Workplace Interventions for Reducing Sitting at Work. *Cochrane Database of Systematic Reviews* **2018**, CD010912, doi:10.1002/14651858.CD010912.pub3.www.cochranelibrary.com.
49. Aittasalo, M.; Livson, M.; Lusa, S.; Romo, A.; Vähä-Ypyä, H.; Tokola, K.; Sievänen, H.; Mänttäri, A.; Vasankari, T. Moving to Business – Changes in Physical Activity and Sedentary Behavior after Multilevel Intervention in Small and Medium-Size Workplaces. *BMC Public Health* **2017**, *17*, 319–333, doi:10.1186/s12889-017-4229-4.

50. Kahneman, D.; Knetsch, J.L.; Thaler, R.H. Anomalies: The Endowment Effect, Loss Aversion, and Status Quo Bias. *The Journal of Economic Perspectives* **1991**, *5*, 193–206.
51. Venema, T.A.G.; Kroese, F.M.; De Ridder, D.T.D. I'm Still Standing: A Longitudinal Study on the Effect of a Default Nudge. *Psychology and Health* **2018**, *33*, 669–681, doi:10.1080/08870446.2017.1385786.
52. Coffeng, J.K.; Boot, C.R.L.; Duijts, S.F.A.; Twisk, J.W.R.; Van Mechelen, W.; Hendriksen, I.J.M. Effectiveness of a Worksite Social & Physical Environment Intervention on Need for Recovery, Physical Activity and Relaxation; Results of a Randomized Controlled Trial. *PLoS ONE* **2014**, *9*, 1–26, doi:10.1371/journal.pone.0114860.
53. Gilson, N.D.; Puig-Ribera, A.; McKenna, J.; Brown, W.J.; Burton, N.W.; Cooke, C.B. Do Walking Strategies to Increase Physical Activity Reduce Reported Sitting in Workplaces: A Randomized Control Trial. *International Journal of Behavioral Nutrition and Physical Activity* **2009**, *6*, 1–7, doi:10.1186/1479-5868-6-43.
54. Lang, J.J.; McNeil, J.; Tremblay, M.S.; Saunders, T.J. Sit Less, Stand More: A Randomized Point-of-Decision Prompt Intervention to Reduce Sedentary Time. *Preventive Medicine* **2015**, *73*, 67–69, doi:10.1016/j.ypmed.2015.01.026.
55. van Nieuw-Amerongen, M.E.; Kremers, S.P.J.; de Vries, N.K.; Kok, G. The Use of Prompts, Increased Accessibility, Visibility, and Aesthetics of the Stairwell to Promote Stair Use in a University Building. *Environment and Behavior* **2011**, *43*, 131–139, doi:10.1177/0013916509341242.
56. Van Houten, R.; Nau, P.A.; Merrigan, M. Reducing Elevator Energy Use: A Comparison of Posted Feedback and Reduced Elevator Convenience. **1981**, *14*, 377–387.
57. Bellicha, A.; Kieusseian, A.; Fontvieille, A.-M.; Tataranni, A.; Charreire, H.; Oppert, J.-M. Stair-Use Interventions in Worksites and Public Settings - A Systematic Review of Effectiveness and External Validity. *Preventive Medicine* **2015**, *70*, 3–13, doi:10.1016/j.ypmed.2014.11.001.
58. Van Calster, L.; Van Hoecke, A.-S.; Octaef, A.; Boen, F. Does a Video Displaying a Stair Climbing Model Increase Stair Use in a Worksite Setting? *Public Health* **2017**, *149*, 11–20, doi:10.1016/j.puhe.2017.04.007.
59. Bellicha, A.; Kieusseian, A.; Fontvieille, A.-M.; Tataranni, A.; Copin, N.; Charreire, H.; Oppert, J.-M. A Multistage Controlled Intervention to Increase Stair Climbing at Work: Effectiveness and Process Evaluation. *International Journal of Behavioral Nutrition and Physical Activity* **2016**, *13*, 47–55, doi:10.1186/s12966-016-0371-0.
60. Jennings, C.A.; Yun, L.; Loitz, C.C.; Lee, E.-Y.; Mummery, W.K. A Systematic Review of Interventions to Increase Stair Use. *American Journal of Preventive Medicine* **2017**, *52*, 106–114, doi:10.1016/j.amepre.2016.08.014.
61. Swenson, T.; Siegel, M. Increasing Stair Use in an Office Worksite through an Interactive Environmental Intervention. *American Journal of Health Promotion : AJHP* **2013**, *27*, 323–9, doi:10.4278/ajhp.120221-QUAN-104.
62. Boutelle, K.N.; Jeffery, R.W.; Nurray, D.M.; Schmitz, M.K.H. Using Signs, Artwork, and Music to Promote Stair Use in a Public Building. *American Journal of Public Health* **2001**, *91*, 2004–2006, doi:10.1016/0002-8223(93)90850-K.
63. Park, A.E.; Zahiri, H.R.; Hallbeck, M.S.; Augenstein, V.; Sutton, E.; Yu, D.; Lowndes, B.R.; Bingener, J. Intraoperative "Micro Breaks" with Targeted Stretching Enhance Surgeon Physical Function and Mental Focus: A Multicenter Cohort Study. *Annals of Surgery* **2017**, *265*, 340–346, doi:10.1097/SLA.0000000000001665.
64. Conger, J.C.; Conger, A.J.; Costanzo, P.R.; Wright, K.L. The Effect of Social Cues on the Eating Behavior of Obese and Normal Subjects. *Journal of personality* **1980**, *48*, 258–271.
65. De Luca, R. V; Spigelman, M.N. Effects of Models on Food Intake of Obese and Non-Obese Female College Students. *Canadian Journal of Behavioural Science/Revue canadienne des sciences du comportement* **1979**, *11*, 124–129, doi:10.1037/h0081579.
66. Johnston, L. Behavioral Mimicry and Stigmatization. *Social Cognition* **2002**, *20*, 18–35, doi:10.1521/soco.20.1.18.20944.
67. Hermans, R.C.J.; Larsen, J.K.; Herman, C.P.; Engels, R.C.M.E. Modeling of Palatable Food Intake in Female Young Adults. Effects of Perceived Body Size. *Appetite* **2008**, *51*, 512–518, doi:10.1016/j.appet.2008.03.016.
68. McFerran, B.; Dahl, D.W.; Fitzsimons, G.J.; Morales, A.C. I'll Have What She's Having: Effects of Social Influence and Body Type on the Food Choices of Others. *Journal of Consumer Research* **2010**, *36*, 915–929, doi:10.1086/644611.
69. Cruwys, T.; Bevelander, K.E.; Hermans, R.C.J. Social Modeling of Eating: A Review of When and Why Social Influence Affects Food Intake and Choice. *Appetite* **2015**, *86*, 3–18, doi:10.1016/j.appet.2014.08.035.
70. Swartz, A.M.; Rote, A.E.; Welch, W.A.; Maeda, H.; Hart, L.; Cho, Y.I.; Strath, S.J. Prompts to Disrupt Sitting Time and Increase Physical Activity at Work, 2011–2012. *Preventing Chronic Disease* **2014**, *11*, 1–8.
71. Donath, L.; Faude, O.; Schefer, Y.; Roth, R.; Zahner, L. Repetitive Daily Point of Choice Prompts and Occupational Sit-Stand Transfers, Concentration and Neuromuscular Performance in Office Workers: An RCT. *International Journal of Environmental Research and Public Health* **2015**, *12*, 4340–4353, doi:10.3390/ijerph120404340.
72. Evans, R.E.; Fawole, H.O.; Sheriff, S.A.; Dall, P.M.; Grant, P.M.; Ryan, C.G. Point-of-Choice Prompts to Reduce Sitting Time at Work: A Randomized Trial. *American Journal of Preventive Medicine* **2012**, *43*, 293–297, doi:10.1016/j.amepre.2012.05.010.

73. Pedersen, S.J.; Cooley, P.D.; Mainsbridge, C. An E-Health Intervention Designed to Increase Workday Energy Expenditure by Reducing Prolonged Occupational Sitting Habits. *Work* **2014**, *49*, 289–295, doi:10.3233/WOR-131644.
74. Cooley, D.; Pedersen, S.; Mainsbridge, C. Assessment of the Impact of a Workplace Intervention to Reduce Prolonged Occupational Sitting Time. *Qualitative Health Research* **2014**, *24*, 90–101, doi:10.1177/1049732313513503.
75. Heerwagen, J.H.; Heubach, J.G.; Montgomery, J.; Weimer, W.C. Environmental Design, Work, and Well Being: Managing Occupational Stress through Changes in the Workplace Environment. *AAOHN journal : official journal of the American Association of Occupational Health Nurses* **1995**, *43*, 458–68.
