# Supplementary material for: Choice Architecture Cueing to Healthier Dietary Choices and Physical Activity at the Workplace: Implementation and Feasibility Evaluation
Source: Nutrients. 2021 Oct 14;13(10):3592. doi: 10.3390/nu13103592 (PMC8538928; doi:10.3390/nu13103592)
Supplement: Supplementary file 1 [file nutrients-13-03592-s001.zip › Supplementary material S2.pdf]

Supplementary material 2

Framework for assessing the quality of implementation in the StopDia at Work intervention

Table 1. Implementation quality assessment framework with implementation settings, implemented strategies, and essential elements of and evaluation scale for each strategy.

Target	Setting	Practical strategy	Essential element	Implementation quality		
				**Successful (2 p)**	**Imperfect (1 p)**	**Failed (0 p)**
Nutrition	***Food provision***					
	Meetings	1. Enable healthy choices	Healthy foods and beverages are available.	Healthy options are available by rule or frequently.	Healthy options are available occasionally.	No healthy options are available.
	Cafeteria	2. Widen selection	More options become available in the cafeteria that meet the nutritional criteria of the Heart Symbol.	The offering broadens with all or most of the agreed products, and these products are available most of the time.	The offering broadens with some but not all agreed products and/or the agreed products are available only occasionally.	The offering does not broaden with new Heart Symbol products.
	Meetings	3. Replace with better alternatives	Energy dense and nutritionally poor foods and beverages are replaced with nutritionally better options.	Healthier options replace unhealthy options by rule or frequently.	Healthier options replace unhealthy options occasionally.	Healthier options do not replace unhealthy options.
	Cafeteria	4. Increase visibility and proximity	Options that meet the nutritional criteria of the Heart Symbol are easy to notice and access.	All or most of the agreed changes to product placement materialise most of the time.	Some but not all agreed changes to product placement materialise and/or the changes materialise only occasionally.	Agreed changes to product placement do not materialise.
		5. Decrease visibility and proximity	Less healthy options are less easy to notice and access.	All or most of the agreed changes to product placement materialise most of the time.	Some but not all agreed changes to product placement materialise and/or the changes materialise only occasionally.	Agreed changes to product placement do not materialise.
	Meetings	6. Increase convenience	Fruit and vegetable are served ready to eat.	Fruit and vegetable are served ready to eat by rule or frequently.	Fruit and vegetable are served ready to eat occasionally.	Fruit and vegetable are not served ready to eat.
	Cafeteria	7. Increase perceived variety	Salad components are not mixed but available in separate serving dishes.	Salad components are available in separate serving dishes mostly as planned.	Salad components are available in separate serving dishes only occasionally.	Salad components are not available in separate serving dishes.
		8. Use smaller serving dishes	Less healthy food options are available in smaller serving dishes.	Agreed options are available in smaller serving dishes mostly as planned.	Agreed options are available in smaller serving dishes only occasionally.	Agreed options are not available in smaller serving dishes.
		9. Use smaller serving utensils	Less healthy food options have smaller serving utensils.	Agreed options have smaller serving utensils.	Some but not all agreed options have smaller serving utensils and/or the options have smaller utensils only occasionally.	Agreed options do not have smaller serving utensils.

Target	Setting	Practical strategy	Essential element	Implementation quality		
				**Successful (2 p)**	**Imperfect (1 p)**	**Failed (0 p)**
	Meetings	10. Use smaller serving sizes	Energy dense and nutritionally poor foods and beverages are available in smaller servings.	Unhealthy options are available in smaller servings by rule or frequently.	Unhealthy options are available in smaller servings occasionally.	Unhealthy options are not available in smaller servings.
	Cafeteria	11. One plate-policy	Separate bread and salad plates are moved out of sight to guide employees choose one large plate for lunch.	Separate bread and salad plates are out of sight most of the time.	Separate bread and salad plates are out of sight only occasionally.	Separate bread and salad plates are in sight.
		12. Point-of-choice prompts	The Heart Symbol adjoins and indicates products that meet its nutritional criteria.	Heart Symbol labels are in use and placed correctly most of the time.	Heart Symbol labels are in use and/or placed correctly only occasionally.	Heart Symbol labels are not in use or they are placed incorrectly.
		13. Prime for better choices	Follow the heart-posters are saliently on view at restaurant entrance and/or at the beginning of the buffet.	Follow the heart-posters are on view as planned.	Follow the heart-posters are on view but not as planned.	Follow the heart-posters are not on view.
	Drinking water					
	Personal workstation	14. Facilitate and remind of drinking water	Employees receive personal, reusable water bottles.	Each employee received a water bottle.	Not every employee received a water bottle.	No employee received a water bottle.
	Packed lunches and snacks					
	Coffee rooms	15. Encourage smart packed lunches	Packed Lunch of the Week-recipes are saliently on view and easily accessible to all employees, and the recipes change regularly.	The recipes are easily noticeable and change mostly every 1-2 weeks, except during holidays. Print recipe cards are on display in their cardboard stands or spread, for example, on coffee room table.	• The recipes are not on view or are not easily noticeable.<br>• The recipes change only occasionally.<br>• All recipes of the year are mixed up, for example, in a cardboard box.	Implementation never took off or it ceased at an early phase of the intervention.
		16. Encourage provision of fruit at work.	Materials needed for founding a fruit crew (i.e., the A4-sized instructions/enrolment form and the fruit box) are saliently on view and easily accessible to all employees.	Both the instructions/enrolment form and the fruit box are easily noticeable. Alternatively, the employer provides fruit to employees and dedicated employees organize the fruit offering regularly.	• The instructions/enrolment form is on view with or without the A2-sized campaign poster, but the fruit box is missing.<br>• The fruit box and the A2-sized campaign poster are on view, but the instructions/enrolment form is missing.<br>• The materials are not easily noticeable.	• Only the A2-sized poster or the fruit box is on view.<br>• The materials were never on view as planned or they were taken away at an early phase of the intervention.
Physical activity	***Time spent sitting***					
	Common environments	17. Enable active sitting	Wobble chairs or balance cushions are available and enable more active sitting.	Wobble chairs or balance cushions are available to all employees.	Wobble chairs or balance cushions are available to some but not all employees.	Wobble chairs or balance cushions are not available.
	Stair use					

Target	Setting	Practical strategy	Essential element	Implementation quality		
				Successful (2 p)	Imperfect (1 p)	Failed (0 p)
	Elevator, stairs	18. Enhance stairwell visibility	Footprints saliently guide to stairwell from the point-of-choice between the stairs and the elevator.	Footprints are easily noticeable and placed correctly.	Footprints are not easily noticeable and/or placed incorrectly.	Footprints are not in use.
	Elevator	19. Prompt choosing the stairs	Stickers with the StopDia project logo are saliently on view on elevator doors, next to the call buttons, or in their immediacy.	The StopDia logos are easily noticeable and placed correctly.	The StopDia logos are not easily noticeable and/or placed incorrectly.	The StopDia logos are not in use.
	Movement breaks					
	Common environments	20. Prompt context-specific movement	Where employees pause and movement is possible, Flex!-movement posters are saliently on view. The posters reach all employees independent of where they work.	The posters are located as planned, easily noticeable, and accessible to all employees.	The posters are located where performing suggested movements is not possible, the posters are not easy to notice, and/or they are sparsely on view considering the size of the workplace.	The posters were not laid out or they were taken away at an early phase of the intervention.
		21. Enable movement with exercise equipment	Exercise equipment is available for employees to use.	Exercise equipment is available to all employees.	Exercise equipment is available to some but not all employees.	Exercise equipment is not available.
		22. Prompt movement with exercise equipment	Exercise equipment is saliently on view and easily accessible for all employees.	Exercise equipment is easily noticeable and accessible as planned. Additionally, the equipment can be accompanied with a corresponding Flex!-movement poster (see #20).	Exercise equipment has moved from its place, but is still available.	Exercise equipment is not available or placed completely out of sight, for example, in a cupboard.
	Personal workstation	23. Prompt movement with automatic reminders	Employees have access to an application that prompts to move at pre-set intervals.	The application is available to all employees.	The application is available to some but not all employees.	The application is available to no employee.
